# Cytokinocytes: the diverse contribution of keratinocytes to immune responses in skin

**DOI:** 10.1172/jci.insight.142067

**Published:** 2020-10-15

**Authors:** Yanyun Jiang, Lam C. Tsoi, Allison C. Billi, Nicole L. Ward, Paul W. Harms, Chang Zeng, Emanual Maverakis, J. Michelle Kahlenberg, Johann E. Gudjonsson

**Affiliations:** 1 Department of Dermatology, University of Michigan, Ann Arbor, Michigan, USA.; 2 Department of Dermatology, Peking Union Medical College Hospital, Chinese Academy of Medical Sciences and Peking Union Medical College, Beijing, China.; 3 Department of Computational Medicine and Bioinformatics and Department of Biostatistics, University of Michigan Medical School, Ann Arbor, Michigan, USA.; 4 Department of Nutrition and Department of Dermatology, Case Western Reserve University, Cleveland, Ohio, USA.; 5 Department of Pathology, University of Michigan, Ann Arbor, Michigan, USA.; 6 Department of Dermatology, University of California, Davis, Sacramento, California, USA.; 7 Division of Rheumatology, Department of Internal Medicine, University of Michigan, Michigan, USA.; 8 A. Alfred Taubman Medical Research Institute, Michigan, USA.

## Abstract

The skin serves as the primary interface between our body and the external environment and acts as a barrier against entry of physical agents, chemicals, and microbes. Keratinocytes make up the main cellular constitute of the outermost layer of the skin, contributing to the formation of the epidermis, and they are crucial for maintaining the integrity of this barrier. Beyond serving as a physical barrier component, keratinocytes actively participate in maintaining tissue homeostasis, shaping, amplifying, and regulating immune responses in skin. Keratinocytes act as sentinels, continuously monitoring changes in the environment, and, through microbial sensing, stretch, or other physical stimuli, can initiate a broad range of inflammatory responses via secretion of various cytokines, chemokines, and growth factors. This diverse function of keratinocytes contributes to the highly variable clinical manifestation of skin immune responses. In this Review, we highlight the highly diverse functions of epidermal keratinocytes and their contribution to various immune-mediated skin diseases.

## Introduction

Skin, as the major interface with the external environment, serves as the first line of defense against microorganisms, physical agents, and chemicals. Cutaneous homeostasis and skin defense are maintained by dynamic cellular communication between different cell types in the skin through interactions with various mediators, including growth factors, chemokines, and cytokines. Keratinocytes are the major cell type of the epidermis and are no longer considered components of a passive physical barrier. Indeed, keratinocytes are highly active sentinel cells, expressing a range of pathogen-associated molecular pattern (PAMP) receptors and cytokine receptors; they are capable of responding (as well as producing) a wide variety of cytokines, chemokines, and growth factors. Thus, keratinocytes contribute to many, if not all, inflammatory skin disorders. Based on the diverse range of inflammatory mediators produced by keratinocytes, they have justly been referred to as “cytokinocytes”(1). In this Review, we highlight the role of keratinocytes in physiologic immune responses and as central players in the pathophysiology of inflammatory skin disorders.

## Structure and function of the skin

**Figure 1:**
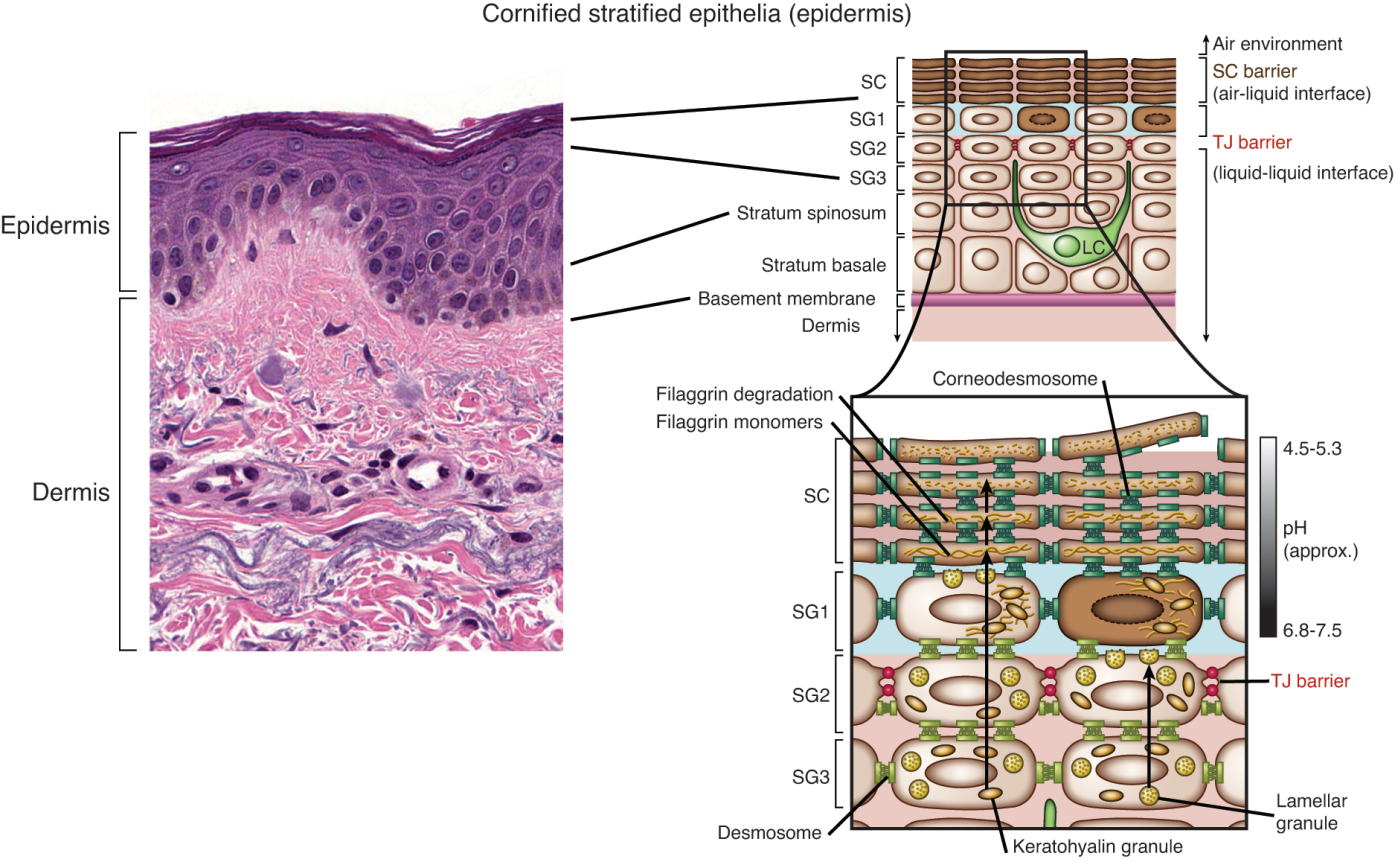
Histology of the skin and the epidermis. The epidermis is a dynamic tissue where keratinocytes exist in various stages of differentiation. Stem cells reside in the basal layer (stratum basale) of the epidermis and move through the subsequent layers with different progression of differentiation in the spinous layer (stratum spinosum), before expressing keratohyalin granules in the granular layer (stratum granulosum [SG]) and terminal differentiation in the stratum corneum (SC). Hematoxylin and eosin staining is shown in the image on the left (original magnification, ×200). Langerhans cells (LCs) reside within the epidermis as part of their surveillance function. In the stratum granulosum keratinocyte synthesis keratohyalin granules contain filaggrin monomers and lamellar granules contain lipids. Tight junctions are expressed in the granular layer. A pH gradient is maintained from the granular layer to the top of the stratum corneum, maintaining a pH of 4.5–5.3 at the surface of the skin. Adapted with permission from *Journal of Clinical Investigation* (158).

Mammalian skin comprises different layers, providing functions for barrier integrity and host defense. The outermost layer, the epidermis, contains no blood vessels and is dependent on the dermis, where blood vessels and lymphatics reside, to provide access to nutrients and waste disposal. The epidermis consists primarily of keratinocytes and can be divided into four layers based on keratinocyte cell morphology and position. These layers, from deep to superficial, are the basal cell layer (stratum basale), the spinous or squamous cell layer (stratum spinosum), the granular cell layer (stratum granulosum), and the cornified or horny cell layer (stratum corneum) (Figure 1). The epidermis on the palms of the hand and soles of the feet has an extra layer, named the clear or translucent layer (stratum lucidum), that can be seen between the granular and the cornified layer (2). Keratinocytes originate in the basal layer. The epidermis also harbors other minor cell populations, including melanocytes, Langerhans cells, and occasionally lymphocytes and Merkel cells, of which the latter are thought to act as mechanoreceptors for light touch sensation. The intermediate layer of the skin is the dermis, which consists of collagen, elastic tissue, and other extracellular components as well as blood and lymphatic vessels, nerve endings, hair follicles, and eccrine glands. Fibroblasts are the primary cell type within the dermis. Additionally, immune cells, such as mast cells, macrophages, and small numbers of lymphocytes, also reside in the dermis. The dermis comprises the thickest skin layer, providing flexibility and strength, and aiding thermal regulation and sensation of various stimuli. The innermost layer is the hypodermis or subcutis, which stores fat as an energy reserve for the body, protects the inner organs as a shock absorber, and has an important role in thermoregulation.

### Physical composition and differentiation of the epidermis.

The epidermis serves vital functions, including limiting passive water loss from the body, preventing absorption of chemicals from the outside environment, and preventing microorganism entry. These functions rely on terminal differentiation, culminating in the formation of the cornified layer (3, 4). An integral part of differentiation is the calcium gradient of the epidermis that peaks in the granular layer and contributes to the regulation of keratinocyte differentiation (5). Each epidermis layer has distinct morphologic and biochemical features, suggesting different roles in skin barrier function. Keratinocytes in the basal layer are responsible for the regeneration of the epidermis and are characterized by expression of keratins 5 and 14 (KRT5 and KRT14). KRT1 and KRT10 are more prominently expressed in the spinous layer, which also has an abundance of desmosomes that provide tissue stability to resist physical trauma. In the granular layer of the skin, keratinocytes synthesize keratohyalin granules, which contain the proteins profilaggrin and loricrin, along with lipid-enriched lamellar bodies. The granular layer is the site of a series of transformative processes required for development of the cornified layer, which involves loss of keratinocyte nucleus and the cytoplasm, aggregation of keratins into microfibrils, and replacement of cell membrane with a cell envelope (CE) assembled by cross-linked proteins. The main structural components of the CE keratohyalin granules contain loricrin, filaggrin, and involucrin, which are cross-linked by transglutaminase enzymes. Subsequently, the extracellular surface of the CE is extruded by lipids, from the lamellar bodies, to form the cornified lipid envelope (6). The CE and cornified lipid envelope are major contributors to skin barrier function. Tight junctions in the granular layer limit the passage of solutes and water in the space between the keratinocytes (and loss to the environment) and, therefore, play an essential role in epidermal barrier formation (7). A contributor to this process is the aryl hydrocarbon receptor (AHR), a ligand-activated transcription factor that is abundantly expressed in all skin cells (8); it also contributes to this process and is critical for both epidermal differentiation (9) and modulation of inflammatory responses (10).

Keratinocytes also contribute to the formation of epidermal appendages, including hair follicles, sebaceous glands, and eccrine glands. Hair follicles originate from the epidermis during embryogenesis as downward-projecting epithelial buds, forming units with associated structures, including sebaceous glands and arrector pili muscle. Sebaceous glands produce sebum that contributes to skin acidity and impedes entry of microbes. Sebum also has antibacterial effects at the external skin surface, further contributing to skin immune defense (11). Eccrine glands have transport sweat via secretory coils in the deep dermis that open up on the surface of the skin, consisting of two layers of tubular epithelium. In addition to thermoregulation, eccrine glands also secrete several antimicrobial peptides (AMPs), such as dermcidin, which serve to control skin flora and prevent skin infections (12).

### AMP production.

AMPs are small cationic and amphipathic molecules that are expressed constitutively in the skin and provide broad protection against bacteria, fungi, and viruses (13). AMP expression can be upregulated by microbial stimuli, proinflammatory cytokines (i.e., IL-1β, TNF, IL-17, and IL-22) (14), as part of keratinocyte differentiation (15), or during wound healing (16). These peptides play a vital role in normal skin homeostasis and epidermal resistance to infections (17). Keratinocytes are major producers of AMPs, including defensins, cathelicidin (LL37), psoriasin (S100A7), and the antimicrobial protein RNase7 (18). AMPs are important for skin homeostasis, as demonstrated by diseases such as atopic dermatitis (AD) and psoriasis. Patients with AD are susceptible to recurrent microbial infections due to a relative decrease in expression of cathelicidin and β-defensins (19), while, conversely, patients with psoriasis are more resistant to skin infections due to high AMP levels (20). In addition to direct antimicrobial killing, AMPs recruit immune cells and modulate cytokine and chemokine production (13, 21, 22). For example, human β-defensins and LL-37 promote recruitment of leukocytes, including neutrophils, T cells, mast cells, and monocytes, to infection sites (13, 23). LL-37 can also promote epithelial cell proliferation (24). Furthermore, antiviral proteins (AVPs), a subset of AMPs, directly antagonize viral infections. The majority of AVPs are induced and amplified by IFNs, but not exclusively, as is seen with IL-27 induction of AVPs in keratinocytes in the setting of Zika virus infection (25).

### Contribution of pH to epidermal defenses.

Human skin pH is tightly regulated and maintained at an acidic pH, ranging from pH 4–6. Sebum contributes to epidermal surface acidity, which helps regulate cutaneous microbial flora and protect against infection. For example, the acidity of the skin is inhospitable for many pathogenic microorganisms, such as *Staphylococcus aureus,* but is permissive for commensal bacteria, such as *Staphylococcus epidermidis* (26). In addition to sebum, the barrier protein filaggrin also contributes to low skin pH due to the high content of acidic amino acids among its breakdown products. Accordingly, decreases in filaggrin, as is seen in AD, result skin pH elevation, thereby contributing to predisposition for *S*. *aureus* carriage in patients with AD (27). Finally, pH influences skin barrier function by regulating enzymes of ceramide metabolism as well as function of various proteases (28).

### Abnormal epidermal barrier function.

AD is a chronic inflammatory skin disease characterized by altered keratinocyte differentiation in epidermal compartments, including the basal and spinous layers (6). A 2006 study identified filaggrin gene mutations as a predisposing factor for AD (29). Filaggrin is expressed in the granular layer as a precursor protein, profilaggrin. Filaggrin is important for keratinization and is eventually metabolized into natural moisturizing factor, which maintains skin hydration by promoting water retention within the stratum corneum (30). AD has also been associated with decreases in other skin barrier proteins, including filaggrin-2, corneodesmosin, desmoglein-1, desmocollin-1, and transglutaminase-3 (31). Loss of filaggrin and abnormal keratinocyte differentiation allows for a more permissive skin environment that promotes entry of irritants, allergens, and microbes that may evoke immune responses, which then further promote progressive weakening of the epidermal barrier, a phenomenon prominent in chronic skin diseases, such as AD, and an emerging concept in autoimmune skin disease, such as cutaneous lupus erythematosus (CLE) (32).

The epidermis reacts to repetitive trauma or inflammatory stimuli by increased keratinocyte proliferation, resulting in epidermal thickening. The thickened epidermis presents clinically as “lichenification” or “psoriasiform hyperplasia.” This thickening is seen in psoriasis and promotes amplification of inflammatory responses through a feed-forward mechanism (33). We recently demonstrated that IRAK2, a member of the signaling complex downstream of IL-1/IL-36, is important in this process as it primes the atopic and psoriatic epidermis for inflammation through promotion of altered epidermal differentiation in a highly coordinated and regulated fashion (our unpublished observations).

## Keratinocytes as sentinels in skin immune defense

Keratinocytes are able to detect a broad range of PAMPs (34), such as LPS, lipopeptides, β-glucans, and single- and double-stranded RNA (dsRNA) and DNA, present on Gram-positive and Gram-negative bacteria, fungi, and viral species through various pattern recognition receptors (PRRs). Keratinocytes express several different types of PRRs, including TLRs, nucleotide-binding oligomerization domain-like receptors (NLRs), RIG-I–like receptors (RLRs), and C-type lectin receptors (CLRs) (Table 1) (35–43). Spatial distribution these receptors is distinct and dependent on epidermis layer. For example, our recent single-cell analysis of human skin revealed that mRNA expression of RNA and DNA sensors (i.e., *TLR3*, *STING*, *MDA5*) is higher in the basal layers of the epidermis, likely accounting for antiviral responses being most prominent in these layers (our unpublished data).

Keratinocytes express a variety of TLRs (TLR1–TLR6, TLR9, and TLR10, which is human specific) (44). TLR1, TLR2, TLR4, TLR5, and TLR6 are found on the cell membrane, while TLR3, TLR7, TLR8, and TLR9 reside in intracellular compartments, such as endosomes and lysosomes (44). Recognition of their cognitive ligand by TLRs on keratinocytes initiates immune responses via activation of downstream signaling cascades involving the MYD88 complex, with the exception of TLR3 (35), and production of chemokines, cytokines, and AMPs. TLR2 recognizes components expressed by bacteria that can trigger the production of proinflammatory cytokines, such as IL-8, TNF, and IL-6 (45, 46). TLR4 recognizes LPS. TLR5 recognizes bacterial flagellin, which can induce NF-κB translocation and IL-8 secretion (47). Intracellular TLRs detect nucleic acid from viruses or bacteria that have been broken down and taken into cells (40). For example, TLR3 binds dsRNA that originates from viruses. TLR9 activation on keratinocytes by CpG-methylated DNA selectively induces production of CXCL9, CXCL10, and type I IFNs (48).

NLRs are intracellular sensors of bacterial infection and cellular damage and can be divided into three subfamilies: the NOD-containing, the NLRP, and the NLRC families (49). NOD1 and NOD2 are the best-characterized members of the NOD-containing subfamily and are functional in human keratinocytes (50), where they respond to bacterial peptidoglycan fragments (50), such as γ-glutamyl-diaminopimelic acid and muramyl dipeptide. NOD1 induces expression and secretion of CXCL8 (also known as IL-8) in keratinocytes (51). Other NLRP members are expressed in keratinocytes and activate inflammatory caspases (49).For example, dsRNA-mediated NLRP3 inflammasome activation can result in IL-1β and IL-18 release (52). Other NLRP3 activators, including UVB-induced DNA damage, house dust mite, and pesticides, have been reported in keratinocytes (53–55). Recently published data has suggested that NLRP1 is the predominant NOD family member in keratinocytes and has a crucial role in UVB sensing and IL-1β and -18 secretion by human keratinocytes (49).

RLRs are crucial for host antiviral defense and sense dsRNA, resulting in type I IFN production. RIG-I (also known as DDX58) is a cytosolic PRR critical for recognizing viral antigens and activating type I IFN responses. Other RLRs include melanoma differentiation-associated protein 5 (MDA5) and LGP2 (42). Keratinocytes efficiently respond to viral dsRNA by expressing RIG-I and MDA5 and promoting IRF3 activation (56). Other cytosolic DNA sensor-linked PRPs include absent in melanoma 2 (AIM2) and STING. AIM2 recognizes cytosolic DNA and triggers inflammasome activation and IL-1β production in keratinocytes, thereby contributing to psoriasis pathogenesis (57). STING also acts as a key component in DNA-mediated innate immunity, which can induce production of type I IFNs (58).

The C-type lectin domain family 7 member A, or Dectin-1, is a receptor that recognizes fungal antigens, such as β-glucan, and *Mycobacterium ulcerans* has recently been shown to induce Dectin-1 expression in human keratinocytes in a TLR2-dependent manner (59). Additionally, curdlan, a water-insoluble linear β-1,3-glucan, enhances keratinocyte proliferation and migration through Dectin-1 binding; therefore, therapeutic targeting of Dectin-1 in keratinocytes has been proposed for therapeutic use in wound healing (43).

DOCK8, a guanine nucleotide exchange factor for Rho GTPases, plays a critical role in regulating immune cell trafficking and microbial-host interactions (60). Thus, DOCK8 deficiency in humans is associated with atopy and a notable increase in cutaneous viral colonization and infection (61).

Keratinocytes may also alter their surface phenotype to communicate with their surroundings. For example, upon IFN-γ exposure, keratinocytes have been shown to express MHC II molecules on their surface and act as an antigen-presenting cells for CD4^+^ and CD8^+^ memory T cells, consequently inducing functional responses (62). A recent study showed that keratinocyte-intrinsic MHC class II expression controls commensal-induced Th1, but not Th17, responses, reflecting the important role of keratinocytes in regulating the host-microbiota communication (63).

Beyond microbes, keratinocytes are also continuously exposed to, and sense, mechanical stimuli such as pressure and stretch. The effects of mechanical stretching on keratinocyte immune response have been investigated in detail. Treatment of keratinocytes with mechanical stimulation activates the secretion of inflammatory cytokines (IL-1α, IL-6, IL-23, and TNF) and chemokines (CXCL1 and CCL20) (64–66). Other studies have reported that mechanical stretching promotes keratinocyte proliferation and inhibits keratinocyte differentiation (65, 67, 68).

## Keratinocytes: source and target of inflammatory mediators

**Figure 2:**
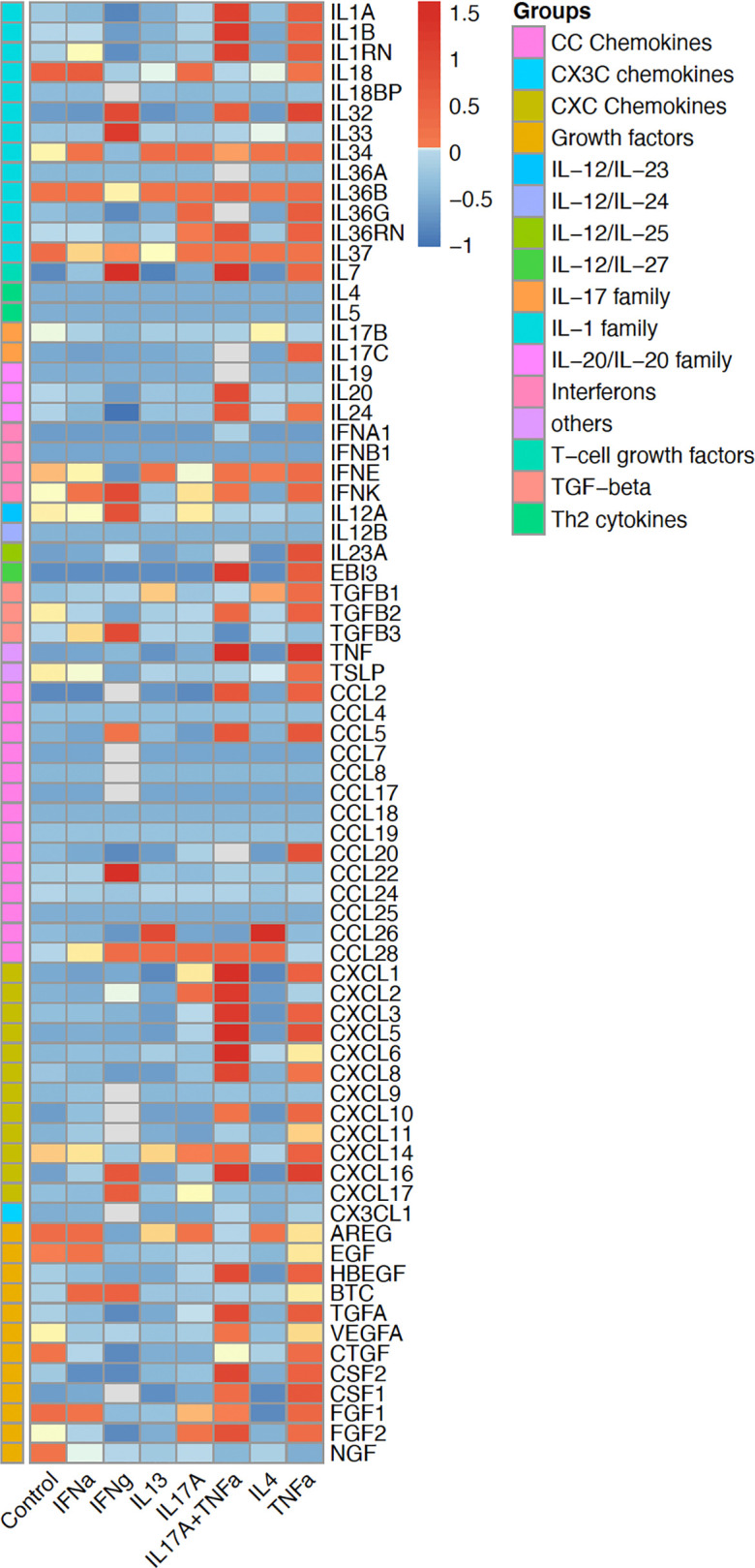
Expression of various cytokines and chemokines in keratinocytes. The heatmap shows the induction of cytokines, chemokines, and various growth factors, via RNA sequencing, in human keratinocytes under various inflammatory conditions, including stimulation with IFN-α, IFN-γ, IL-13, IL-17A, TNF-α, IL-4, and IL-17A^+^ TNF-α. Keratinocytes have a dynamic response depending on the type of stimulation. The color gradient represents expression levels after normalization.

Keratinocytes express and secrete a broad range of cytokines that can affect and amplify inflammatory responses, induce keratinocyte proliferation, and promote migration of leukocytes into skin (50, 69) (Figure 2). Here, we present a short overview of several key cytokines and cytokine families implicated in keratinocyte immune responses.

### The IL-1 cytokine family — IL-1, IL-36, and beyond.

The IL-1 family of cytokines is divided into three subfamilies: IL-1, IL-18, and IL-36 (70). Keratinocytes constitutively produce IL-1α and IL-1β, which bind to the same receptor complex and have similar biologic activity. Upon PRR stimulation, keratinocytes release IL-1 to initiate a rapid immune response, leading to expression of other cytokines, including IL-6, IL-8, and TNF (71). IL-1 also promotes keratinocyte proliferation (72). IL-1 subfamily member IL-33 is constitutively expressed in keratinocytes and signals via the ST2 receptor, resulting in Th2 cytokine induction (73). The IL-33/ST2 axis has been implicated in several skin diseases, including AD and psoriasis (74).

IL-18 is synthesized as an inactive precursor similar to IL-1β that requires processing into the mature molecule by the action of IL-1β converting enzyme (75). IL-18 is produced by keratinocytes and can augment Th1 responses in the presence of IL-12 by inducing IFN-γ production (75). IL-18 also enhances IFN-γ–induced production of chemokines (CXCL9, CXCL10, and CXCL11) and MHC class I expression on keratinocytes (76). IL-1 family member IL-37 can interact with the IL-18 receptor, and IL-37 overexpression in human keratinocytes inhibits the production of CXCL8, IL-6, and S100A7, suggesting that IL-37 may play an immunosuppressive role in psoriasis pathogenesis (77).

IL-36 subfamily cytokine expression is induced in keratinocytes by microbes and cytokines, including TNF, IL-17, and IL-22 (50, 78). IL-36 induces production of other proinflammatory cytokines and AMPs as well as its own expression in an autocrine manner (78, 79). IL-38 is the newest and least-characterized member of this family and is expressed in epithelia, including skin; however, its role in skin inflammation remains to be defined.

### TNF.

TNF has pleiotropic effects on a variety of processes, including cell proliferation, differentiation, apoptosis, and inflammation, and exerts its biological effects through binding to either of the 2 membrane-bound TNF receptors, TNFR1 or TNFR2. Keratinocytes both produce (80) and respond (81) to TNF through TNFR1. TNFR1 signaling leads to activation of several downstream transcription factors, including NF-κB, AP-1, and CCAAT enhancer–binding protein-β (C/EPβ) (82). This activation leads to expression of proinflammatory cytokines, chemokines, and adhesion molecules, such as IL-1, IL-6, CXCL8, CCL20, and ICAM-1 (83). Furthermore, TNF potently synergizes with other cytokines, such as IL-17A (84), IL-17C (85) and others, to amplify immune responses in keratinocytes (82).

### IL-6 and IL-31.

IL-6 is expressed by human keratinocytes and is reported to be overexpressed in many inflammatory skin diseases, such as psoriasis (79, 86), CLE (87), and lichen planus (88). IL-6 expression is induced by multiple stimuli, including UVB radiation, TLR stimulation, and various proinflammatory cytokines (89). IL-6 promotes keratinocyte proliferation and is prominent in diseases associated with epidermal hyperplasia and tissue injury (90). IL-6 family member IL-31 is primarily secreted by activated T cells. The IL-31 receptor, a heterodimer of IL-31 receptor α chain (IL-31RA) and oncostatin M receptor β chain (OSMR β), is expressed by immune cells and keratinocytes (91). IL-31 upregulates the expression levels of various chemokines and AMPs in keratinocytes. In addition, IL-31 downregulates filaggrin expression and has been implicated in AD pathogenesis (91). Consistent with a role in AD, overexpression of IL-31 in mouse skin induces severe pruritus and dermatitis (92).

### IL-12 and IL-23.

IL-12 and IL-23 influence the adaptive immune system, promoting Th1 and Th17 responses, respectively. IL-12 is a heterodimeric cytokine formed by p40 and p35 subunits, which are expressed and released by human keratinocytes (93). IL-12 is involved in promoting Th1 responses and inducing IFN-γ production by T cells and NK cells (94). The common p40 and the unique IL-23p19 subunits constitute IL-23, which signals via a receptor complex composed of the IL-23R and IL-12R-β1 receptor units. Recently, keratinocytes were determined to express and secrete IL-23 (95). Keratinocyte-derived IL-23 can promote chronic IL-17–skewed skin inflammation and is regulated by TNF and Wiskott-Aldrich syndrome protein (N-WASP) (96). While keratinocytes secrete less IL-23 per cell than other cell types (such as DCs), the sheer number of keratinocytes in skin make them an important IL-23 source in inflammatory skin conditions.

### IL-20 cytokines.

The IL-20 cytokine family consists of IL-19, IL-20, IL-22, IL-24, and IL-26. Among these, IL-19, IL-20, and IL-24 share the same receptor complex (IL-20RB/IL-20RA), while IL-20 and IL-24 can also bind the IL-20RB/IL-22RA1 heterodimer (97). Keratinocytes are a major target of IL-20 cytokines, where these cytokines act in an autocrine manner. IL-20 cytokines are induced by other leukocyte-derived proinflammatory cytokines, such as IL-22, IL-1, IL-17A, and TNF (98). IL-20 cytokines induce keratinocyte proliferation and promote production of inflammatory and immunomodulatory mediators through activation of STAT3 (99).

### IL-7, IL-15, and TSLP.

Keratinocytes are an important source of IL-7 and IL-15, which function as T cell growth factors. Keratinocyte-derived IL-7 promotes adhesion of epidermal T cells to laminin-5 (100) in the basement membrane. In psoriasis, IL-7 promotes interactions between keratinocytes and T lymphocytes (101), while IL-15 enhances proliferation and activity NK cells and T cells. In addition, both IL-7 and IL-15 play important roles in the generation and maintenance of memory T cells (102). Notably, hair follicle keratinocytes are prominent sources of IL-7 and IL-15 and facilitate skin-resident memory T cell homeostasis (103). Thymic stromal lymphopoietin (TSLP) is an IL-7–like cytokine that was initially discovered as a stimulator of B cell development from a murine thymic stromal cell line (104). TSLP binds to a heterodimeric receptor composed of the TSLP receptor chain (TSLPR) and the IL-7 receptor α chain (105). TSLP is expressed by keratinocytes in response to certain microbial products, trauma, or inflammatory cytokines (106), and keratinocyte-derived TSLP helps promote Th2 cell differentiation and inflammation in allergic diseases (107).

### IFNs.

IFNs were first identified for their antiviral activities and have been classified into three major types: type I IFNs (IFN-α, IFN-β, IFN-ɛ, IFN-κ, IFN-τ, and IFN-ω), type II IFNs (IFN-γ), and type III IFNs (IFN-λ1, IFN-λ2, and IFN-λ3) (108). IFN-κ is the predominant type I IFN expressed by keratinocytes, particularly in the basal layer, and is prominent in skin lesions of CLE (109), although its expression has also been described in psoriasis (110). IFN-β is also expressed by keratinocytes after stimulation by UV light and TLR3 stimulation (111). Expression of type III IFNs has been described in keratinocytes and is reported to be increased in cutaneous lupus lesions (112). Keratinocytes do not express IFN-γ.

### Growth factors.

Keratinocytes secrete a large number of various growth factors, including EGF family factors, TGF-β, FGF family members, GM-CSF, PDGF, and VEGF. Beyond its profibrotic effects, TGF-β controls keratinocyte proliferation and differentiation and accelerates wound-healing processes (113). TGF-α, a member of EGF family, promotes keratinocyte migration and proliferation, suggesting a role in reepithelialization (114). Keratinocyte-derived GM-CSF stimulates keratinocyte proliferation in vivo and is essential for Langerhans cell maturation (115). Keratinocytes also secret VEGF, which promotes angiogenesis and endothelial cell migration, and PDGF, which enhances fibroblast proliferation and ECM component production (116). Nerve growth factor (NGF), a neurotrophic peptide, can be synthesized and released by human keratinocytes, promotes keratinocyte proliferation, and is increased in psoriatic keratinocytes (117).

### Cytokine receptor expression in keratinocytes.

Keratinocytes express a wide range of cytokine receptors capable of binding to cytokines released by either other epidermal cells or infiltrating leukocytes (Table 2) (71, 91, 97, 118–121), consistent with keratinocytes as a frequent target for inflammatory processes in skin. Thus, activation of IL-17 and IL-22 receptors on keratinocytes amplifies inflammatory responses in diseases such as psoriasis (44, 122). Keratinocytes also express receptors for Th2 cytokines, IL-4 and IL-13 (88), IL-1 and IL-36 (123, 124), and type I and type II IFNs (120). Therefore, keratinocytes are able to respond to almost the entire spectrum of polarizing inflammatory signals, whether they are Th1-, Th2-, Th17- or autoinflammatory-driven responses. Interestingly, specific layers of the epidermis may respond differently to particular inflammatory signals. In particular, spatial differences in immune responses can be seen for type I IFN responses, which are primarily seen in the basal keratinocytes (109), while IL-17 responses are most prominent in the spinous layer of the epidermis (our unpublished observation).

### Keratinocyte-derived chemokines.

Keratinocytes are also a relevant source of chemokines and chemokine receptors (17) (see Figure 2 and Table 2). Chemokines are small, secreted proteins that have chemotactic activity. They are divided into four main subfamilies, CC, CXC, C, and CX3C chemokines, which exert their biological effects via interaction with their cognate cell-surface chemokine receptors (125). Keratinocytes synthesize many chemokines that attract distinct cells types into the skin during inflammatory or immune responses depending on the specific chemokines expressed (81). For example, keratinocyte-derived chemokine ligands CXCL9 and CXCL10 bind CXCR3 and CCL27 to CCR10 to help T cells traffic into the skin in diseases such as psoriasis (126). Similarly, keratinocytes secrete CCR4 ligand CCL17, thereby promoting Th2 cell recruitment into AD skin (127). Keratinocytes upregulate CXCL1 and mediate neutrophil trafficking into skin (17, 128, 129).

### Keratinocyte proteases and protease inhibitors.

Keratinocytes express and secrete various proteases and protease inhibitors. Under homeostatic conditions these proteases regulate processes related to epidermal differentiation, but under inflammatory conditions they may contribute to both initiation and termination of inflammatory responses. Cathepsin S, a lysosomal cysteine protease that takes part in the degradation of damaged or unwanted proteins, has been identified as one of the major IL-36γ–activating proteases in the skin (130), thereby initiating and amplifying skin-associated inflammatory responses. Recently, Billi et al. determined that epidermal kallikrein-related peptidase 6 (KLK6), a secreted serine protease, promotes development of psoriasis and psoriatic arthritis-like joint inflammation via protease-activated receptor 1–dependent (PAR1-dependent) signaling (131).

Given their potent biological role in the skin, activity of these proteases is tightly regulated by protease inhibitors. Keratinocytes express and secrete a wide variety of such inhibitors, including serine protease inhibitors (SERPINs), which are believed to be involved in the pathophysiology of various inflammatory skin diseases (132). For example, recently described loss-of-function variants in *SERPINA3* associated with development of generalized pustular psoriasis. Thus, decreased production of serpin A3 weakens inhibition of neutrophil serine protease cathepsin G and therefore leads to unrestrained activity of cathepsin G and subsequent activation of IL-36 family cytokines (133, 134). Moreover, SERPIN Kazal-type 5 (SPINK5, also known as LEKTI) is prominently expressed in epithelial and mucosal tissues. *SPINK5* mutations result in activation of proteases, such as KLK5, KLK7, and KLK14, that produce skin desquamation and were identified as the cause of Netherton syndrome, which is characterized by congenital ichthyosis with hair shaft anomalies and atopic manifestations (135, 136).

### Complement protein expression in keratinocytes.

Keratinocytes are a source of complement proteins, thereby providing another line of immunologic defense in the skin. Keratinocytes express several complement system components, including C3, C4, factor B, factor H, factor I, complement receptors (CR1, cC1qR, C5aR1, and CR2); cell-bound complement regulators (MCP, DAF, and CD59); and terminal complement components (C5–C9) (137, 138). Complement production in keratinocytes is regulated by a variety of cytokines. C3 synthesis is regulated by TNF, IFN-γ, and IL-1α. Factor B expression is induced by IFN-γ, IL-1α, and IL-6 (139), and IL-22 and TNF synergistically induce production of complement C1r and C1s in primary human keratinocytes (140). IFN-γ and IFN-α have also been shown to upregulate complement fragment-3a receptor (C3aR) on keratinocytes (141).

## Contribution of keratinocytes in inflammatory conditions

### Psoriasis.

Psoriasis is a chronic inflammatory skin disease characterized by keratinocyte hyperproliferation and immune cell infiltration in the dermis and epidermis (142). Accumulating evidence indicates that keratinocytes may play a greater role in psoriasis pathogenesis than previously thought. Thus, keratinocyte release of the AMP LL-37 may be involved in triggering psoriasis (143). LL-37 conjugates with self-DNA and self-RNA fragments, leading to activation of TLR9 in plasmacytoid DCs to produce IFN-α and trigger psoriasis (143) and subsequent activation of myeloid DCs and migration to skin-draining lymph nodes where they interact with naive T cells to promote expansion and differentiation of Th17, Th22, and Th1 lymphocyte subsets (144, 145).

Cytokines such as IL-17A upregulate additional cytokines, chemokines, and AMPs in keratinocytes (146) and, with IL-22, promote keratinocyte proliferation (44, 147). Keratinocyte-derived cytokines such as IL-36 influence DC function, induce secretion of proinflammatory cytokines, and promote T cell proliferation (148). IL-6 promotes Th17 cell differentiation and keratinocyte proliferation (86), but, interestingly, anti–IL-6 treatment has been shown to be ineffective for psoriasis (149). Moreover, keratinocytes produce chemokines that facilitate immune cell recruitment to the skin. CXCL8 acts as a chemoattractant for neutrophils, while CCL2, CCL5, and CXCL10 recruit monocytes and Th1 cells. CCL20 interacts with CCR6 and acts as a chemoattractant for Th17 cells and DCs (150), thereby creating a self-sustaining cycle of inflammation.

### AD.

In most cases, AD is considered to be an allergen-driven disease due to decreased filaggrin barrier function and presence of antigen-presenting cells and effector Th2 cells (151). However, as the main constituents of the epidermis, keratinocytes are active participants and amplify immune responses associated with AD development (152). Keratinocyte-derived AD-associated inflammatory molecules have been identified, including TSLP, that are highly expressed in both acute and chronic lesions. TSLP activates DCs to produce Th2-recruiting chemokines and triggers production of Th2 cytokines from CD4^+^ T cells (153). Moreover, skin-specific TSLP induction in transgenic mice causes an AD-like phenotype, with development of eczematous lesions, Th2 cell infiltration, and elevated serum IgE levels (154). Notably, keratinocytes from patients with AD exhibit an intrinsically abnormal chemokine production profile characterized by high expression of CCL5, CCL27, CCL17, CCL22, and CCL18 (155).

### Other inflammatory diseases and wound healing.

CLE is a chronic inflammatory disease frequently associated with systemic lupus erythematosus. Keratinocyte apoptosis is considered to play a role in CLE pathogenesis (83, 156), and increased type I IFNs are considered an important pathway in its pathogenesis (83). In particular, keratinocyte- produced IFN-κ promotes IFN responsiveness and photosensitivity in keratinocytes in CLE (109). In addition, type I IFNs (IFN-α and IFN-κ) are reported to amplify IL-6 production by lupus keratinocytes following TLR exposure and UV irradiation (87).

Wound healing is a dynamic and complex process that requires communication and coordination among diverse cell populations. Among them, keratinocytes play a significant role. Thus, during the wound-healing process, migration and proliferation of keratinocytes are promoted to reepithelialize the wound surface. Growth factors from the EGF, FGF, and TGF-β families promote keratinocyte proliferation in wound healing (114). In addition, keratinocytes also contribute and produce cytokines, including IL-1, IL-6, and TNF, that can promote keratinocyte migration (157).

## Summary

**Figure 3:**
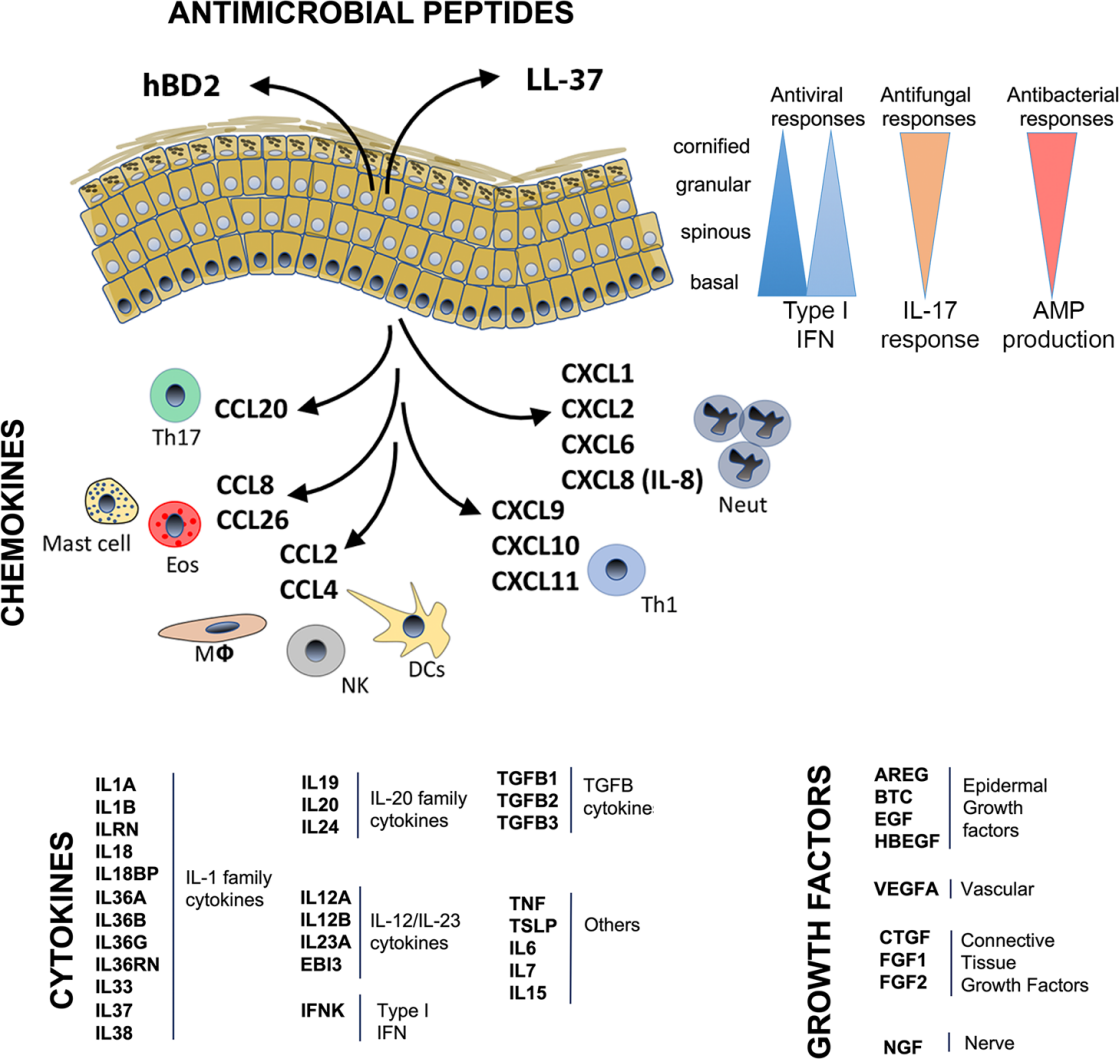
Diverse role of keratinocytes in inflammatory responses. Keratinocytes express different microbial sensors and cytokine receptors, with highly variable expression in different layers of the epidermis. This likely accounts for type I IFN responses (antiviral) being most prominent in the basal layer of the epidermis but anti–IL-17 (antifungal) or antibacterial responses (as measured by expression of AMPs encoding genes) dominating in the top layer. In addition, keratinocytes express and secrete a variety of proinflammatory cytokines, including the IL-1 family members, IL20 family members, IL-12/IL-23 cytokines, type I IFN, IFN-κ, and various others. In addition, keratinocytes secrete a variety of growth factors that influence epidermis growth and differentiation, vascular growth, fibroblast proliferation, and nerves. The various chemokines secreted by keratinocytes drive influx of various types of inflammatory cell subsets depending on the chemokine signal.

Keratinocytes are a highly dynamic cell type with functional roles that extend far beyond their participation as components of the physical barrier of the skin. Keratinocytes are equipped with molecular sensors to detect nearly any type of microbe, from simple viruses to bacteria to fungi and parasites. Furthermore, keratinocytes express and secrete a wide variety of proinflammatory cytokines, chemokines, and growth factors, leaving them capable of directing and activating almost any type of polarized immune response, including Th1, Th2, Th17, and various autoinflammatory responses (Figure 3). These dynamic responses explain the diverse range of clinical presentation of inflammatory skin diseases that far exceeds that of any other organ system. Future discoveries will extend our understanding of the role of the keratinocyte and its contribution to disease pathogenesis and will facilitate development of new treatments for cutaneous disorders.

## Figures and Tables

**Table 1:**
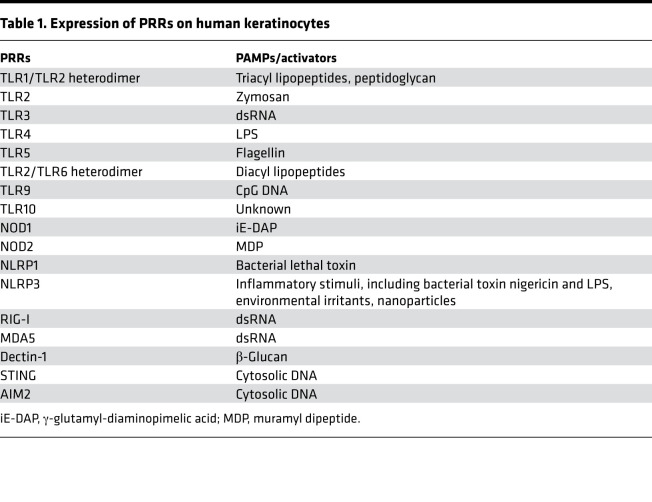
Expression of PRRs on human keratinocytes

**Table 2:**
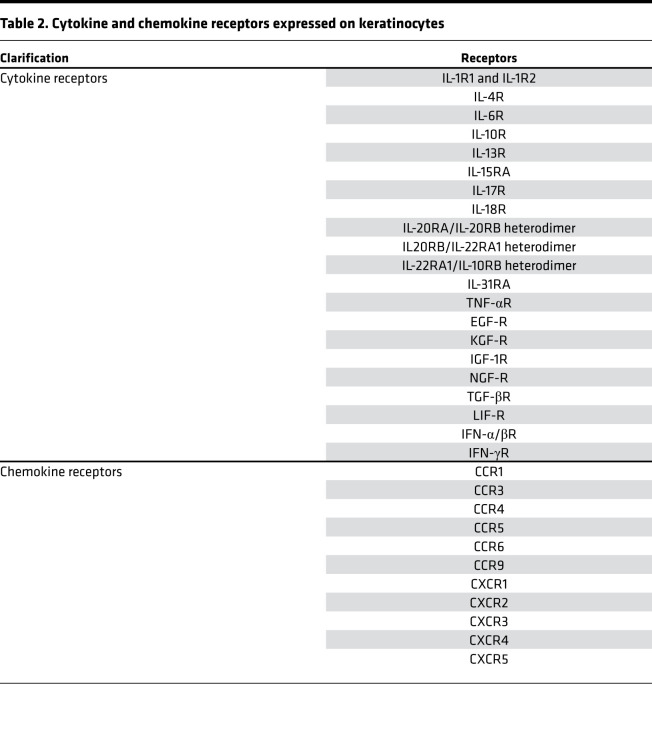
Cytokine and chemokine receptors expressed on keratinocytes

## Version 1 10/15/2020

Electronic publication

## References

[1] Singh A, Morris RJ (2012). Innate immunity and the regulation and mobilization of keratinocyte stem cells: are the old players playing a new game?. Exp Dermatol.

[2] Kanitakis J (2002). Anatomy, histology and immunohistochemistry of normal human skin. Eur J Dermatol.

[3] Rosso JD, Zeichner J, Alexis A, Cohen D, Berson D (2016). Understanding the epidermal barrier in healthy and compromised skin: clinically relevant information for the dermatology practitioner: proceedings of an expert panel roundtable meeting. J Clin Aesthet Dermatol.

[4] Wickett RR, Visscher MO (2006). Structure and function of the epidermal barrier. Am J Infect Control.

[5] Bikle DD, Xie Z, Tu CL (2012). Calcium regulation of keratinocyte differentiation. Expert Rev Endocrinol Metab.

[6] Goleva E, Berdyshev E, Leung DY (2019). Epithelial barrier repair and prevention of allergy. J Clin Invest.

[7] Bäsler K, Brandner JM (2017). Tight junctions in skin inflammation. Pflugers Arch.

[8] Esser C, Bargen I, Weighardt H, Haarmann-Stemmann T, Krutmann J (2013). Functions of the aryl hydrocarbon receptor in the skin. Semin Immunopathol.

[9] van den Bogaard EH (2015). Genetic and pharmacological analysis identifies a physiological role for the AHR in epidermal differentiation. J Invest Dermatol.

[10] Di Meglio P (2014). Activation of the aryl hydrocarbon receptor dampens the severity of inflammatory skin conditions. Immunity.

[11] Gallo RL, Nakatsuji T (2011). Microbial symbiosis with the innate immune defense system of the skin. J Invest Dermatol.

[12] Cui CY, Schlessinger D (2015). Eccrine sweat gland development and sweat secretion. Exp Dermatol.

[13] Clausen ML, Agner T (2016). Antimicrobial peptides, infections and the skin barrier. Curr Probl Dermatol.

[14] Schittek B (2011). The antimicrobial skin barrier in patients with atopic dermatitis. Curr Probl Dermatol.

[15] Harder J, Meyer-Hoffert U, Wehkamp K, Schwichtenberg L, Schröder JM (2004). Differential gene induction of human beta-defensins (hBD-1, -2, -3, and -4) in keratinocytes is inhibited by retinoic acid. J Invest Dermatol.

[16] Sørensen OE, Cowland JB, Theilgaard-Mönch K, Liu L, Ganz T, Borregaard N (2003). Wound healing and expression of antimicrobial peptides/polypeptides in human keratinocytes, a consequence of common growth factors. J Immunol.

[17] Matejuk A (2018). Skin immunity. Arch Immunol Ther Exp (Warsz).

[18] Suter MM, Schulze K, Bergman W, Welle M, Roosje P, Müller EJ (2009). The keratinocyte in epidermal renewal and defence. Vet Dermatol.

[19] Ong PY (2002). Endogenous antimicrobial peptides and skin infections in atopic dermatitis. N Engl J Med.

[20] de Jongh GJ (2005). High expression levels of keratinocyte antimicrobial proteins in psoriasis compared with atopic dermatitis. J Invest Dermatol.

[21] Niyonsaba F (2007). Antimicrobial peptides human beta-defensins stimulate epidermal keratinocyte migration, proliferation and production of proinflammatory cytokines and chemokines. J Invest Dermatol.

[22] Niyonsaba F, Ushio H, Nagaoka I, Okumura K, Ogawa H (2005). The human beta-defensins (-1, -2, -3, -4) and cathelicidin LL-37 induce IL-18 secretion through p38 and ERK MAPK activation in primary human keratinocytes. J Immunol.

[23] Niyonsaba F, Nagaoka I, Ogawa H (2006). Human defensins and cathelicidins in the skin: beyond direct antimicrobial properties. Crit Rev Immunol.

[24] Heilborn JD (2003). The cathelicidin anti-microbial peptide LL-37 is involved in re-epithelialization of human skin wounds and is lacking in chronic ulcer epithelium. J Invest Dermatol.

[25] Kwock JT (2020). IL-27 signaling activates skin cells to induce innate antiviral proteins and protects against Zika virus infection. Sci Adv.

[26] Nguyen AV, Soulika AM (2019). The dynamics of the skin’s immune system. Int J Mol Sci.

[27] Miajlovic H, Fallon PG, Irvine AD, Foster TJ (2010). Effect of filaggrin breakdown products on growth of and protein expression by *Staphylococcus aureus*. J Allergy Clin Immunol.

[28] Proksch E (2018). pH in nature, humans and skin. J Dermatol.

[29] Palmer CN (2006). Common loss-of-function variants of the epidermal barrier protein filaggrin are a major predisposing factor for atopic dermatitis. Nat Genet.

[30] Sandilands A, Sutherland C, Irvine AD, McLean WH (2009). Filaggrin in the frontline: role in skin barrier function and disease. J Cell Sci.

[31] Broccardo CJ (2011). Comparative proteomic profiling of patients with atopic dermatitis based on history of eczema herpeticum infection and *Staphylococcus aureus* colonization. J Allergy Clin Immunol.

[32] Sirobhushanam S (2020). *Staphylococcus aureus* colonization is increased on lupus skin lesions and is promoted by IFN-mediated barrier disruption. J Invest Dermatol.

[33] Hawkes JE, Chan TC, Krueger JG (2017). Psoriasis pathogenesis and the development of novel targeted immune therapies. J Allergy Clin Immunol.

[34] Coates M, Blanchard S, MacLeod AS (2018). Innate antimicrobial immunity in the skin: A protective barrier against bacteria, viruses, and fungi. PLoS Pathog.

[35] Miller LS (2008). Toll-like receptors in skin. Adv Dermatol.

[36] Ermertcan AT, Öztürk F, Gündüz K (2011). Toll-like receptors and skin. J Eur Acad Dermatol Venereol.

[37] Terhorst D, Kalali BN, Ollert M, Ring J, Mempel M (2010). The role of toll-like receptors in host defenses and their relevance to dermatologic diseases. Am J Clin Dermatol.

[38] Hari A, Flach TL, Shi Y, Mydlarski PR (2010). Toll-like receptors: role in dermatological disease. Mediators Inflamm.

[39] Lebre MC (2007). Human keratinocytes express functional Toll-like receptor 3, 4, 5, and 9. J Invest Dermatol.

[40] Oviedo-Boyso J, Bravo-Patiño A, Baizabal-Aguirre VM (2014). Collaborative action of Toll-like and NOD-like receptors as modulators of the inflammatory response to pathogenic bacteria. Mediators Inflamm.

[41] Awad F (2018). Photoaging and skin cancer: is the inflammasome the missing link?. Mech Ageing Dev.

[42] Kawai T, Akira S (2009). The roles of TLRs, RLRs and NLRs in pathogen recognition. Int Immunol.

[43] van den Berg LM, Zijlstra-Willems EM, Richters CD, Ulrich MM, Geijtenbeek TB (2014). Dectin-1 activation induces proliferation and migration of human keratinocytes enhancing wound re-epithelialization. Cell Immunol.

[44] Klicznik MM, Szenes-Nagy AB, Campbell DJ, Gratz IK (2018). Taking the lead - how keratinocytes orchestrate skin T cell immunity. Immunol Lett.

[45] Mempel M (2003). Toll-like receptor expression in human keratinocytes: nuclear factor kappaB controlled gene activation by *Staphylococcus aureus* is Toll-like receptor 2 but not toll-like receptor 4 or platelet activating factor receptor dependent. J Invest Dermatol.

[46] Meisgen F (2014). MiR-146a negatively regulates TLR2-induced inflammatory responses in keratinocytes. J Invest Dermatol.

[47] Köllisch G (2005). Various members of the Toll-like receptor family contribute to the innate immune response of human epidermal keratinocytes. Immunology.

[48] Miller LS, Modlin RL (2007). Human keratinocyte Toll-like receptors promote distinct immune responses. J Invest Dermatol.

[49] Burian M, Yazdi AS (2018). NLRP1 is the key inflammasome in primary human keratinocytes. J Invest Dermatol.

[50] Bitschar K, Wolz C, Krismer B, Peschel A, Schittek B (2017). Keratinocytes as sensors and central players in the immune defense against *Staphylococcus aureus* in the skin. J Dermatol Sci.

[51] Harder J, Núñez G (2009). Functional expression of the intracellular pattern recognition receptor NOD1 in human keratinocytes. J Invest Dermatol.

[52] Dai X, Tohyama M, Murakami M, Sayama K (2017). Epidermal keratinocytes sense dsRNA via the NLRP3 inflammasome, mediating interleukin (IL)-1β and IL-18 release. Exp Dermatol.

[53] Hasegawa T, Nakashima M, Suzuki Y (2016). Nuclear DNA damage-triggered NLRP3 inflammasome activation promotes UVB-induced inflammatory responses in human keratinocytes. Biochem Biophys Res Commun.

[54] Dai X (2011). Mite allergen is a danger signal for the skin via activation of inflammasome in keratinocytes. J Allergy Clin Immunol.

[55] Jang Y (2015). Chlorpyrifos induces NLRP3 inflammasome and pyroptosis/apoptosis via mitochondrial oxidative stress in human keratinocyte HaCaT cells. Toxicology.

[56] Kalali BN (2008). Double-stranded RNA induces an antiviral defense status in epidermal keratinocytes through TLR3-, PKR-, and MDA5/RIG-I-mediated differential signaling. J Immunol.

[57] Dombrowski Y (2011). Cytosolic DNA triggers inflammasome activation in keratinocytes in psoriatic lesions. Sci Transl Med.

[58] Zhu Y, An X, Zhang X, Qiao Y, Zheng T, Li X (2019). STING: a master regulator in the cancer-immunity cycle. Mol Cancer.

[59] Lee HM (2009). Innate immune responses to Mycobacterium ulcerans via toll-like receptors and dectin-1 in human keratinocytes. Cell Microbiol.

[60] Kunimura K, Uruno T, Fukui Y (2020). DOCK family proteins: key players in immune surveillance mechanisms. Int Immunol.

[61] Tirosh O (2018). Expanded skin virome in DOCK8-deficient patients. Nat Med.

[62] Black AP (2007). Human keratinocyte induction of rapid effector function in antigen-specific memory CD4+ and CD8+ T cells. Eur J Immunol.

[63] Tamoutounour S (2019). Keratinocyte-intrinsic MHCII expression controls microbiota-induced Th1 cell responses. Proc Natl Acad Sci USA.

[64] Oh S, Chung H, Chang S, Lee SH, Seok SH, Lee H (2019). Effect of mechanical stretch on the DNCB-induced proinflammatory cytokine secretion in human keratinocytes. Sci Rep.

[65] Qiao P (2019). Mechanical stretch exacerbates psoriasis by stimulating keratinocyte proliferation and cytokine production. J Invest Dermatol.

[66] Lee RT, Briggs WH, Cheng GC, Rossiter HB, Libby P, Kupper T (1997). Mechanical deformation promotes secretion of IL-1 alpha and IL-1 receptor antagonist. J Immunol.

[67] Yano S, Komine M, Fujimoto M, Okochi H, Tamaki K (2004). Mechanical stretching in vitro regulates signal transduction pathways and cellular proliferation in human epidermal keratinocytes. J Invest Dermatol.

[68] Topczewska JM, Ledwon JK, Vaca EE, Gosain AK (2019). Mechanical stretching stimulates growth of the basal layer and rete ridges in the epidermis. J Tissue Eng Regen Med.

[69] Coondoo A (2012). The role of cytokines in the pathomechanism of cutaneous disorders. Indian J Dermatol.

[70] Garlanda C, Dinarello CA, Mantovani A (2013). The interleukin-1 family: back to the future. Immunity.

[71] Uchi H, Terao H, Koga T, Furue M (2000). Cytokines and chemokines in the epidermis. J Dermatol Sci.

[72] Kondo S (1999). The roles of keratinocyte-derived cytokines in the epidermis and their possible responses to UVA-irradiation. J Investig Dermatol Symp Proc.

[73] Schmitz J (2005). IL-33, an interleukin-1-like cytokine that signals via the IL-1 receptor-related protein ST2 and induces T helper type 2-associated cytokines. Immunity.

[74] Imai Y (2019). Interleukin-33 in atopic dermatitis. J Dermatol Sci.

[75] Sanders NL, Mishra A (2016). Role of interleukin-18 in the pathophysiology of allergic diseases. Cytokine Growth Factor Rev.

[76] Kanda N, Shimizu T, Tada Y, Watanabe S (2007). IL-18 enhances IFN-gamma-induced production of CXCL9, CXCL10, and CXCL11 in human keratinocytes. Eur J Immunol.

[77] Teng X (2014). IL-37 ameliorates the inflammatory process in psoriasis by suppressing proinflammatory cytokine production. J Immunol.

[78] Carrier Y (2011). Inter-regulation of Th17 cytokines and the IL-36 cytokines in vitro and in vivo: implications in psoriasis pathogenesis. J Invest Dermatol.

[79] Towne JE, Sims JE (2012). IL-36 in psoriasis. Curr Opin Pharmacol.

[80] Köck A (1990). Human keratinocytes are a source for tumor necrosis factor alpha: evidence for synthesis and release upon stimulation with endotoxin or ultraviolet light. J Exp Med.

[81] Tüzün Y, Antonov M, Dolar N, Wolf R (2007). Keratinocyte cytokine and chemokine receptors. Dermatol Clin.

[82] Banno T, Gazel A, Blumenberg M (2004). Effects of tumor necrosis factor-alpha (TNF alpha) in epidermal keratinocytes revealed using global transcriptional profiling. J Biol Chem.

[83] Robinson ES, Werth VP (2015). The role of cytokines in the pathogenesis of cutaneous lupus erythematosus. Cytokine.

[84] Chiricozzi A (2011). Integrative responses to IL-17 and TNF-α in human keratinocytes account for key inflammatory pathogenic circuits in psoriasis. J Invest Dermatol.

[85] Johnston A (2013). Keratinocyte overexpression of IL-17C promotes psoriasiform skin inflammation. J Immunol.

[86] Grossman RM (1989). Interleukin 6 is expressed in high levels in psoriatic skin and stimulates proliferation of cultured human keratinocytes. Proc Natl Acad Sci USA.

[87] Stannard JN (2017). Lupus skin Is primed for IL-6 inflammatory responses through a keratinocyte-mediated autocrine type I interferon loop. J Invest Dermatol.

[88] Gröne A (2002). Keratinocytes and cytokines. Vet Immunol Immunopathol.

[89] Hunter CA, Jones SA (2015). IL-6 as a keystone cytokine in health and disease. Nat Immunol.

[90] Hernández-Quintero M, Kuri-Harcuch W, González Robles A, Castro-Muñozledo F (2006). Interleukin-6 promotes human epidermal keratinocyte proliferation and keratin cytoskeleton reorganization in culture. Cell Tissue Res.

[91] Nakashima C, Otsuka A, Kabashima K (2018). Interleukin-31 and interleukin-31 receptor: New therapeutic targets for atopic dermatitis. Exp Dermatol.

[92] Dillon SR (2004). Interleukin 31, a cytokine produced by activated T cells, induces dermatitis in mice. Nat Immunol.

[93] Aragane Y (1994). IL-12 is expressed and released by human keratinocytes and epidermoid carcinoma cell lines. J Immunol.

[94] Trinchieri G (2003). Interleukin-12 and the regulation of innate resistance and adaptive immunity. Nat Rev Immunol.

[95] Piskin G, Sylva-Steenland RM, Bos JD, Teunissen MB (2006). In vitro and in situ expression of IL-23 by keratinocytes in healthy skin and psoriasis lesions: enhanced expression in psoriatic skin. J Immunol.

[96] Li H (2018). Epigenetic control of IL-23 expression in keratinocytes is important for chronic skin inflammation. Nat Commun.

[97] Eidenschenk C, Rutz S, Liesenfeld O, Ouyang W (2014). Role of IL-22 in microbial host defense. Curr Top Microbiol Immunol.

[98] Witte E (2014). IL-19 is a component of the pathogenetic IL-23/IL-17 cascade in psoriasis. J Invest Dermatol.

[99] Sa SM (2007). The effects of IL-20 subfamily cytokines on reconstituted human epidermis suggest potential roles in cutaneous innate defense and pathogenic adaptive immunity in psoriasis. J Immunol.

[100] Wagner LA (1999). The keratinocyte-derived cytokine IL-7 increases adhesion of the epidermal T cell subset to the skin basement membrane protein laminin-5. Eur J Immunol.

[101] Szepietowski JC, Bielicka E, Nockowski P, Noworolska A, Wasik F (2000). Increased interleukin-7 levels in the sera of psoriatic patients: lack of correlations with interleukin-6 levels and disease intensity. Clin Exp Dermatol.

[102] Lanzavecchia A, Sallusto F (2005). Understanding the generation and function of memory T cell subsets. Curr Opin Immunol.

[103] Adachi T (2015). Hair follicle-derived IL-7 and IL-15 mediate skin-resident memory T cell homeostasis and lymphoma. Nat Med.

[104] Friend SL, Hosier S, Nelson A, Foxworthe D, Williams DE, Farr A (1994). A thymic stromal cell line supports in vitro development of surface IgM+ B cells and produces a novel growth factor affecting B and T lineage cells. Exp Hematol.

[105] Park LS (2000). Cloning of the murine thymic stromal lymphopoietin (TSLP) receptor: Formation of a functional heteromeric complex requires interleukin 7 receptor. J Exp Med.

[106] Allakhverdi Z (2007). Thymic stromal lymphopoietin is released by human epithelial cells in response to microbes, trauma, or inflammation and potently activates mast cells. J Exp Med.

[107] Indra AK (2013). Epidermal TSLP: a trigger factor for pathogenesis of atopic dermatitis. Expert Rev Proteomics.

[108] Xi Y, Day SL, Jackson RJ, Ranasinghe C (2012). Role of novel type I interferon epsilon in viral infection and mucosal immunity. Mucosal Immunol.

[109] Sarkar MK (2018). Photosensitivity and type I IFN responses in cutaneous lupus are driven by epidermal-derived interferon kappa. Ann Rheum Dis.

[110] Li Y (2019). Interferon kappa is up-regulated in psoriasis and it up-regulates psoriasis-associated cytokines in vivo. Clin Cosmet Investig Dermatol.

[111] Fujisawa H, Kondo S, Wang B, Shivji GM, Sauder DN (1997). The expression and modulation of IFN-alpha and IFN-beta in human keratinocytes. J Interferon Cytokine Res.

[112] Zahn S (2011). Evidence for a pathophysiological role of keratinocyte-derived type III interferon (IFNλ) in cutaneous lupus erythematosus. J Invest Dermatol.

[113] Coondoo A (2011). Cytokines in dermatology - a basic overview. Indian J Dermatol.

[114] Barrientos S, Stojadinovic O, Golinko MS, Brem H, Tomic-Canic M (2008). Growth factors and cytokines in wound healing. Wound Repair Regen.

[115] Feliciani C, Gupta AK, Sauder DN (1996). Keratinocytes and cytokine/growth factors. Crit Rev Oral Biol Med.

[116] Santoro MM, Gaudino G (2005). Cellular and molecular facets of keratinocyte reepithelization during wound healing. Exp Cell Res.

[117] Raychaudhuri SP, Jiang WY, Farber EM (1998). Psoriatic keratinocytes express high levels of nerve growth factor. Acta Derm Venereol.

[118] Bernard FX (2012). Keratinocytes under fire of proinflammatory cytokines: bona fide innate immune cells involved in the physiopathology of chronic atopic dermatitis and psoriasis. J Allergy (Cairo).

[119] Bouchaud G (2013). Epidermal IL-15Rα acts as an endogenous antagonist of psoriasiform inflammation in mouse and man. J Exp Med.

[120] Esche C, de Benedetto A, Beck LA (2004). Keratinocytes in atopic dermatitis: inflammatory signals. Curr Allergy Asthma Rep.

[121] Behm B, Babilas P, Landthaler M, Schreml S (2012). Cytokines, chemokines and growth factors in wound healing. J Eur Acad Dermatol Venereol.

[122] Liang SC (2006). Interleukin (IL)-22 and IL-17 are coexpressed by Th17 cells and cooperatively enhance expression of antimicrobial peptides. J Exp Med.

[123] Rauschmayr T, Groves RW, Kupper TS (1997). Keratinocyte expression of the type 2 interleukin 1 receptor mediates local and specific inhibition of interleukin 1-mediated inflammation. Proc Natl Acad Sci USA.

[124] Goldstein JD (2020). IL-36 signaling in keratinocytes controls early IL-23 production in psoriasis-like dermatitis. Life Sci Alliance.

[125] Sokol CL, Luster AD (2015). The chemokine system in innate immunity. Cold Spring Harb Perspect Biol.

[126] Rottman JB, Smith TL, Ganley KG, Kikuchi T, Krueger JG (2001). Potential role of the chemokine receptors CXCR3, CCR4, and the integrin alphaEbeta7 in the pathogenesis of psoriasis vulgaris. Lab Invest.

[127] Vestergaard C, Bang K, Gesser B, Yoneyama H, Matsushima K, Larsen CG (2000). A Th2 chemokine, TARC, produced by keratinocytes may recruit CLA+CCR4+ lymphocytes into lesional atopic dermatitis skin. J Invest Dermatol.

[128] Nestle FO, Di Meglio P, Qin JZ, Nickoloff BJ (2009). Skin immune sentinels in health and disease. Nat Rev Immunol.

[129] Li N (2014). Alarmin function of cathelicidin antimicrobial peptide LL37 through IL-36γ induction in human epidermal keratinocytes. J Immunol.

[130] Ainscough JS (2017). Cathepsin S is the major activator of the psoriasis-associated proinflammatory cytokine IL-36γ. Proc Natl Acad Sci USA.

[131] Billi AC (2020). KLK6 expression in skin induces PAR1-mediated psoriasiform dermatitis and inflammatory joint disease. J Clin Invest.

[132] Soualmia F, El Amri C (2018). Serine protease inhibitors to treat inflammation: a patent review (2011-2016). Expert Opin Ther Pat.

[133] Frey S (2020). Rare loss-of-function mutation in SERPINA3 in generalized pustular psoriasis. J Invest Dermatol.

[134] Henry CM, Sullivan GP, Clancy DM, Afonina IS, Kulms D, Martin SJ (2016). Neutrophil-derived proteases escalate inflammation through activation of IL-36 family cytokines. Cell Rep.

[135] Chavanas S (2000). Mutations in SPINK5, encoding a serine protease inhibitor, cause Netherton syndrome. Nat Genet.

[136] Furio L, Hovnanian A (2014). Netherton syndrome: defective kallikrein inhibition in the skin leads to skin inflammation and allergy. Biol Chem.

[137] Giang J, Seelen MAJ, van Doorn MBA, Rissmann R, Prens EP, Damman J (2018). Complement activation in inflammatory skin diseases. Front Immunol.

[138] Timár KK, Dallos A, Kiss M, Husz S, Bos JD, Asghar SS (2007). Expression of terminal complement components by human keratinocytes. Mol Immunol.

[139] Pasch MC, Van Den Bosch NH, Daha MR, Bos JD, Asghar SS (2000). Synthesis of complement components C3 and factor B in human keratinocytes is differentially regulated by cytokines. J Invest Dermatol.

[140] Eyerich S (2011). IL-22 and TNF-α represent a key cytokine combination for epidermal integrity during infection with Candida albicans. Eur J Immunol.

[141] Purwar R (2006). Induction of C3 and CCL2 by C3a in keratinocytes: a novel autocrine amplification loop of inflammatory skin reactions. J Immunol.

[142] Deng Y, Chang C, Lu Q (2016). The inflammatory response in psoriasis: a comprehensive review. Clin Rev Allergy Immunol.

[143] Lande R (2007). Plasmacytoid dendritic cells sense self-DNA coupled with antimicrobial peptide. Nature.

[144] Ganguly D (2009). Self-RNA-antimicrobial peptide complexes activate human dendritic cells through TLR7 and TLR8. J Exp Med.

[145] Di Meglio P, Perera GK, Nestle FO (2011). The multitasking organ: recent insights into skin immune function. Immunity.

[146] Furue M, Furue K, Tsuji G, Nakahara T (2020). Interleukin-17A and keratinocytes in psoriasis. Int J Mol Sci.

[147] Georgescu SR (2019). Advances in understanding the immunological pathways in psoriasis. Int J Mol Sci.

[148] Foster AM (2014). IL-36 promotes myeloid cell infiltration, activation, and inflammatory activity in skin. J Immunol.

[149] Mease PJ (2016). The efficacy and safety of clazakizumab, an anti-interleukin-6 monoclonal antibody, in a phase IIb study of adults with active psoriatic arthritis. Arthritis Rheumatol.

[150] Nedoszytko B, Sokołowska-Wojdyło M, Ruckemann-Dziurdzińska K, Roszkiewicz J, Nowicki RJ (2014). Chemokines and cytokines network in the pathogenesis of the inflammatory skin diseases: atopic dermatitis, psoriasis and skin mastocytosis. Postepy Dermatol Alergol.

[151] Leung DY, Bieber T (2003). Atopic dermatitis. Lancet.

[152] Albanesi C (2010). Keratinocytes in allergic skin diseases. Curr Opin Allergy Clin Immunol.

[153] Soumelis V (2002). Human epithelial cells trigger dendritic cell mediated allergic inflammation by producing TSLP. Nat Immunol.

[154] Yoo J (2005). Spontaneous atopic dermatitis in mice expressing an inducible thymic stromal lymphopoietin transgene specifically in the skin. J Exp Med.

[155] Giustizieri ML (2001). Keratinocytes from patients with atopic dermatitis and psoriasis show a distinct chemokine production profile in response to T cell-derived cytokines. J Allergy Clin Immunol.

[156] Baima B, Sticherling M (2001). Apoptosis in different cutaneous manifestations of lupus erythematosus. Br J Dermatol.

[157] Sivamani RK, Garcia MS, Isseroff RR (2007). Wound re-epithelialization: modulating keratinocyte migration in wound healing. Front Biosci.

[158] Kubo A, Nagao K, Amagai M (2012). Epidermal barrier dysfunction and cutaneous sensitization in atopic diseases. J Clin Invest.

